# YAP/TAZ-TEAD activity promotes the malignant transformation of cervical intraepithelial neoplasia through enhancing the characteristics and Warburg effect of cancer stem cells

**DOI:** 10.1007/s10495-023-01935-0

**Published:** 2024-03-29

**Authors:** Shu Li, Xing Li, Yong-bin Yang, Su-fang Wu

**Affiliations:** Department of Obstetrics and Gynecology, Shanghai General Hospital, Shanghai Jiao Tong University School of Medicine, Shanghai, 200080 China

**Keywords:** Cervical intraepithelial neoplasia (CIN), Characteristics of cancer stem cells, Warburg effect, Yes-associated protein (YAP)/transcriptional co-activator with PDZ-binding motif (TAZ), Transcriptional enhanced associate domain (TEAD)

## Abstract

A number of studies have confirmed that Yes-associated protein (YAP)/transcriptional co-activator with PDZ-binding motif (TAZ)-transcriptional enhanced associate domain (TEAD) activity is the driver of cancer development. However, the role and mechanism of the YAP/TAZ-TEAD pathway in cervical intraepithelial neoplasia (CIN) remain to be clarified. Therefore, this study was designed to observe the effect of YAP/TAZ-TEAD activity on the development of CIN and provide new ideas for the diagnosis and treatment of CIN. Firstly, cervical tissues were collected from CIN patients in different stages [CIN grade 1 (CIN1) tissue, CIN grade 2/3 (CIN 2/3) and squamous cell carcinoma (SCC)] and healthy volunteers. Next, the expression levels of YAP, TAZ and TEAD in cervical tissues and cells were observed by immunohistochemistry, qRT-PCR and western blot. Besides, Z172 and Z183 cells were transfected with siRNA-YAP/TAZ (si-YAP/TAZ) and YAP/TAZ overexpression vector (YAP-5SA). Also, Z172 cells were co-transfected with YAP-5SA and si-TEAD2/4. Subsequently, the stemness characteristics, glycolysis level and malignant transformation of cells in each group were observed by sphere-formation assay, commercial kit, MTT, Transwell, scratch experiment, xenotransplantation and western blot.The expression of YAP, TAZ and TEAD increased significantly in cervical cancer tissue and cell line at the stage of CIN2/3 and SCC. When YAP/TAZ was knocked down, the stemness characteristics, glycolysis level and malignant transformation of cancer cells were notably inhibited; while activating YAP/TAZ exhibited a completely opposite result. In addition, activating YAP/TAZ and knocking down the TEAD expression at the same time significant weakened the effect of activated YAP/TAZ signal on precancerous cells and reduced inhibitory effect of knocking down TEAD alone. YAP/TAZ-TEAD signal activates the characteristics and Warburg effect of cancer stem cells, thereby promoting the malignant transformation of CIN.

## Introduction

Globally, cervical cancer is the most common malignant tumor in women, only after breast cancer, lung cancer and colorectal cancer. As early as 2018, some studies estimated that there were as many as 5.7 million new cases of cervical cancer and 311,000 cervical cancer-related deaths each year [1]. The continuous improvement of the early screening program can significant reduce the morbidity and mortality caused by cervical cancer [2]. Moreover, the popularization of HPV vaccine worldwide seems to make people see the hope of avoiding the harm of cervical cancer [3]. However, early screening of cervical cancer is relatively lowly sensitive to cervical intraepithelial neoplasia (CIN) [4]. CIN is the origin of squamous cell carcinoma (SCC, which accounts for about 80% of cervical cancer), but the biomarker antigen (SCC-Ag) commonly used to detect SCC is only effective for advanced cervical cancer [5]. Specifically, the detection rates of cervical cancer at stage III/IV and recurrent malignant tumors were 76.4%, 76.9% and 87%, respectively. Nevertheless, the detection rate of SCC-Ag in early tumors is relatively low [1]. Therefore, it is necessary to find new biomarkers for early detection of cervical cancer, especially for high-grade CIN.

Yes-associated protein (YAP) and transcriptional co-activator with PDZ-binding motif (TAZ) are the core components of the Hippo signaling pathway [6]. The involvement of YAP/TAZ in tumor invasion and metastasis has been fully verified. For instance, a research by Wang et al. proposed that, promoting the mutual activation of YAP/TAZ in breast cancer mice can lead to tumor metastasis to distant organs [7]. Interestingly, Zhang et al. proved that the expression level of YAP/TAZ was positively correlated with the diameter and infiltration of SCC [8]. Also, there are studies that the activation of YAP/TAZ in pyloric stem cells can induce progressive tumors, result in gastric cancer in vivo [9], and promote the progress of CIN [10]. Overall, YAP/TAZ pathway not only is related to the initiation of cancer, but also can regulate the development of cancer. More interestingly, the activation of YAP/TAZ can also affect the metabolic state of cancer cells. A previous research has pointed out that, when YAP/TAZ is fully activated, the glucose uptake and glycolysis rate of cells are increased. In addition, the production of lactic acid of cancer cells with high activity of YAP/TAZ increases relatively, and the pH value of cell culture medium also decreases faster [11]. Hence, some studies have suggested that YAP/TAZ pathway is the root of cancer development [12]. Collectively, the activation of YAP/TAZ has a potential regulatory effect on the progression of CIN.

Transcriptional enhanced associate domain (TEAD) family is the key transcription factor of Hippo pathway in nucleus. On the one hand, TEAD regulates cell growth, proliferation and tissue homeostasis by integrating and coordinating signal transduction pathways such as Hippo, Wnt, transforming growth factor (TGF) β and epidermal growth factor receptor (EGFR). On the other hand, TEAD can play an important role in tumor progression, metastasis, tumor metabolism, immunity and drug resistance through transcription and regulation of KRAS, BRAF, LKB1, NF2 and MYC [13]. In addition, ChIP-seq analysis proved that YAP/TAZ is the main activator of TEAD family, and their interaction can promote the expression of downstream genes [14]. Mori et al. discovered that activation of TEAD1/4 [15] and inhibition of APOBEC3B by VGLL1, a cofactor of TEAD interaction, are important and molecular mechanisms in HPV-induced cervical cancer [16]. Also, these studies have suggested that the TEAD family may play a decisive role in the process of CIN. However, there is no research on the relationship between TEAD and CIN. Therefore, the effect of YAP/TAZ-TEAD activity on CIN was observed in this study to explore new biological targets and therapeutic ideas for the diagnosis and treatment of CIN.

## Materials and methods

### Collection of clinical samples

Cervical tissues of CIN patients who underwent pathological examination in Shanghai General Hospital, Shanghai Jiao Tong University School of Medicine were collected. The collected tissues consisted of normal cervical tissue (Normal), CIN grade 1 (CIN1) tissue, CIN grade 2/3 (CIN 2/3) tissue and SCC tissue. The inclusion criteria were shown as follows: participants (1) were informed and volunteered to participate in this study; (2) were diagnosed as CIN by 2–3 clinicians; (3) conformed to the classification standard established by International Federation of Cervical Pathology and Colposcopy (IFCPC). The exclusion criteria were listed as follows: participants (1) who are pregnant or nursing; (2) with suspected or confirmed invasive SCC and in situ or invasive adenocarcinoma; (3) with immunodeficiency, hepatitis or human immunodeficiency virus infection [17, 18]. This study was approved by the Ethics Review Committee of Shanghai General Hospital, Shanghai Jiao Tong University School of Medicine.

### Cell culture and treatment

Precancerous cervical epithelial cells (Z172, Z183) and cervical cancer cells (SiHa, C-33A) were purchased from National Collection of Authenticated Cell Culture and American Type Culture Collection (ATCC), respectively. Z172 and Z183 were cultured in a special culture medium (IMP-H110-1, Immocell, Xiamen, China) for precancerous cervical epithelial cells supplemented with 10% fetal bovine serum (FBS) and 1% streptomycin. SiHa and C-33A were cultured in a DMEM/F12 medium supplemented with 10% FBS and 1% streptomycin. The culture condition was set as a culture incubator at 37 ℃ and containing 5% CO_2_. After cell passage to the third generation, cell experiments were started. Briefly, Inni-fectin™ PC-sRNA cell transfection reagent was selected to introduce YAP/TAZ siRNA, YAP/TAZ overexpression vector, TEAD2 siRNA and TEAD4 siRNA into Z172 and Z183 cells, which were named as si-YAP/TAZ, YAP-5SA, si-TEAD2, and si-TEAD4, respectively. Besides, TEAD2/4 siRNA and YAP-5SA combined with TEAD2/4 siRNA were transfected into Z172 cells, which were named as si-TEAD2/4 and si-TEAD2/4+YAP-5SA. After 48 h of cell culture, subsequent experiments were carried out.

### Xenograft tumor models

Male BALB/c nude mice aged 4–5 weeks and weighing 18–20 g were selected. They were randomly divided into 3 groups (n = 6/group). Z172 cells (5 × 10^6^) transfected with si-NC, si-YAP/TAZ and YAP/TAZ overexpression were injected subcutaneously into the right dorsal side of male BALB/c nude mice, respectively. After completion of injection, the mice were put back into the cage and allowed to have a free access to food and water. The tumor growth of mice in each group was observed once a week. The length (L) and width (W) of the tumor were measured with a vernier caliper, and the tumor volume was calculated according to the formula (L × W). After 56 days of feeding, the mice were anesthetized with sevoflurane and euthanized. Then, the tumor samples were collected and weighted. All procedures of animal experiments were carried out in accordance with the approval of the Ethics Committee of Shanghai General Hospital, Shanghai Jiao Tong University School of Medicine, and all experiments involving mice were performed in strict compliance with animal welfare standards [19].

### qRT-PCR

Total RNA was extracted from cells and tissues by TRI Reagent (T9424, Sigma, USA). Next, the extracted RNA (2 μg) was reversely transcribed into complementary DNA (cDNA) using StarScript III RT Kit StarScript III Reverse Transcription Kit. Subsequently, 2×RealStar Fast SYBR qPCR Mix (A301, Genstar, Beijing, China) reaction system was adopted for amplification. Cycling conditions were set up in an ABI 7900 real-time fluorescence quantitative PCR system (ABI, USA): initial denaturation (95 °C, 5 min); followed by 40 cycles of denaturation (95 °C, 10 s), annealing (60 °C, 15 s); and a final extension at 72 °C for 5 min. Differences in the amount of total RNA in each sample were calculated and standardized by 2^−∆∆CT^ using GAPDH as an endogenous control. All quantities were expressed as multiples relative to GAPDH expression. The quantitative primers in this study were synthesized by GenScript ProBio (Nanjing), and the primer sequences were shown in Table 1.

**Table 1:** Primer sequences

Gene name	Primer sequences (5′ to 3′)
YAP	F: TGTCCCAGATGAACGTCACAGC
	R: TGGTGGCTGTTTCACTGGAGCA
TAZ	F: GAGGACTTCCTCAGCAATGTGG
	R: CGTTTGTTCCTGGAAGACAGTCA
TEAD1	F: CCTGGCTATCTATCCACCATGTG
	R: TTCTGGTCCTCGTCTTGCCTGT
TEAD2	F: CCGCTACATCAAGCTGAGAACG
	R: GGTTGCCATTGTCTGGAAAGCC
TEAD3	F: AGGCAGTAGATGTGCGCCAGAT
	R: TCCTGGATGGTGCTGTTGAGGT
TEAD4	F: GAAGGTCTGCTCTTTCGGCAAG
	R: GAGGTGCTTGAGCTTGTGGATG
GAPDH	F: GTCTCCTCTGACTTCAACAGCG
	R: ACCACCCTGTTGCTGTAGCCAA

### Western blot

Total protein was extracted from cells and tumor tissues by RIPA lysis buffer (89901, Pierce, USA), and the protein concentration was determined by BCA protein assay (Biyuntian, China). Next, the same amount of protein was subject to 10% sodium dodecyl sulfate polyacrylamide gel electropheresis (SDS-PAGE) and then transferred to polyvinylidene fluoride (PVDF) membrane. The membrane was sealed in 5.0% skimmed milk containing 0.1% Tween-20 (BS100, BioShark, China) for 1 h at ambient temperature, and then incubated with different primary antibodies overnight at 4 °C. The primary antibodies used included anti-YAP (SAB1404823, 1:500, Sigma, USA), anti-TAZ (T4077, 1:500, Sigma, USA), CD44 (1:2000, 15675-1-AP, Proteintech, USA), CD133 (1:2000, 66666-1-Ig, Proteintech, USA), KLF4 (1:1000, 11880-1-AP, Proteintech, USA), Sox2 (1:2000, 66411-1-Ig, Proteintech, USA), TEAD2 (1:1000, 21159-1-AP, Proteintech, USA), TEAD4 (1:1000, 12418-1-AP, Proteintech, USA) or β-actin (1:1000, 81115-1-RR, Proteintech, USA). Subsequently, the membrane was washed with TBST for 3 times, followed by incubation with goat anti-rabbit or anti-mouse IgG coupled to horseradish peroxidase (1:5000, SA00001-1 or SA00001-2, Proteintech, USA) for 1 h at ambient temperature. Next, the membrane was soaked in enhanced chemiluminescence (ECL) solution (310212, Zeta Life, China) for 2 min. Finally, the protein bands were visualized in iBright CL 750 (Thermo Fisher, USA) and analyzed by ImageJ software.

### Sphere-formation assay

The transfected Z172 cells were seeded into a 96-well ultra-low adsorption plate at a density of 200 cells per well and supplemented with a 200 μl of serum-free DMEM/F12 medium containing B12, 20 ng/ml EGF and 20 ng/ml bFGF. After 7 days of culture, the formation of cell spheres (> 70 μm) was observed under an optical microscope (SZ4-DS, SOPTOP, China).

### Determination of glucose, lactic acid content and extracellular acidification rate (ECAR)

The glucose consumption, lactic acid production and extracellular acidification rate (ECAR) of cells in each group were measured according to the operation manuals of glucose uptake colorimetric assay kit (MAK083, Sigma, USA), lactic acid assay kit (MAK064, Sigma, USA) and ECAR assay kit (ECAR, BB-48311, bestbio, China). Among them, the determination of ECAR required to seed cells into a XF24 cell culture plate at a density of 2 × 10^4^ cells/well. After treatment, the culture medium was supplemented with oligomycin (1 μM), 2-deoxyglucose (100 mM) and glucose (10 mM). Upon cell culture at 37 ℃ for 60 min, the cell culture plate was placed in Seahorse XF Extracellular Flux Analyzers (XF24 FluxPak, Agilent, USA), and the ECAR was determined [20].

### Determination of cell viability

To observe the viability of cells, the transfected cells were seeded into a 96-well plate at a density of 1 × 10^4^ cells/well. After different treatments, MTT was added to each well for 4 h of incubation in the dark. Then, the supernatant was removed, and the dissolved dimethyl sulfoxide (DMSO) was added to each well. Subsequently, the optical density at the wavelength of 570 nm was measured by a fully automatic enzyme labeling instrument RNE90002 (REAGEN, USA), and the cell viability was evaluated by the measured optical density.

### Cell invasion assay

To assess cell invasion, cells cultured in a 0.2 ml serum-free DMEM/F12 medium were seeded in the upper chamber of Transwell containing matrigel at a density of 2 × 10^4^ cells/well. A complete medium containing 10% FBS was added to the lower chamber. After incubation at 37 ℃ for 48 h, the cells were stained with crystal violet. Lastly, the number of invaded cells was observed and counted under an optical microscope (SZ4-DS, SOPTOP, China).

### Cell migration assay

To determine the cell migration, the transfected cells were seeded into a 6-well plate at a density of 1 × 10^6^ cells/well. When the cell fusion reached 90%, a straight gap was scratched on the cells with a 100 μl sterile tip. After incubation at 37 ℃ for 48 h, the size of cell gap was observed under an optical microscope (SZ4-DS, SOPTOP, China). After photographing, the cell gap was measured using ImageJ.

### Statistics and analysis

IBM SPSS Statistics V 21.0 was responsible for data analysis. All data in this study were expressed as mean ± standard deviation (SD). Independent t test was employed for comparison between two groups, and one-way ANOVA analysis for comparison among multiple groups. P < 0.05 suggested a significant difference.

## Results

### Correlation of YAP/TAZ with the progression of cervical lesions

**Fig. 1:**
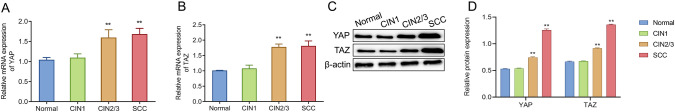
Association between YAP/TAZ and the progression of cervical lesions. **A** and **B** qRT-PCR to detect the mRNA expression levels of YAP (**A**) and TAZ (**B**) in cervical tissues of patients with different stages of cervical lesions; **C** and **D** Western blot to assess the protein expression levels of YAP and TAZ in cervical tissues of patients with different stages of cervical lesions. **P < 0.01, vs normal. *YAP* Yes-associated protein; *TAZ* transcriptional co-activator with PDZ-binding motif

To observe the correlation between YAP/TAZ and the progression of cervical lesions, cervical tissues were collected from CIN patients at different stages (including CIN1/2/3 and SCC). According to the results of qRT-PCR, compared with the Normal group, the expression levels of YAP and TAZ were significantly increased in cervical tissues of CIN2/3 group and SCC group; however, the expression levels of YAP and TAZ between the CIN1 group and normal group were not significantly different (Fig. 1A, B). Besides, the protein expression levels of YAP and TAZ were also evaluated. Undoubtedly, the outcomes of western blot further proved that, compared with the Normal group, the protein expression levels of YAP and TAZ were markedly up-regulated in the CIN2/3 and SCC groups while had no change in the CIN1 group (Fig. 1C, D). Collectively, the expression level of YAP/TAZ changed with the progress of CIN.

### Up-regulated expression of YAP/TAZ in cervical cancer cell lines

**Fig. 2:**
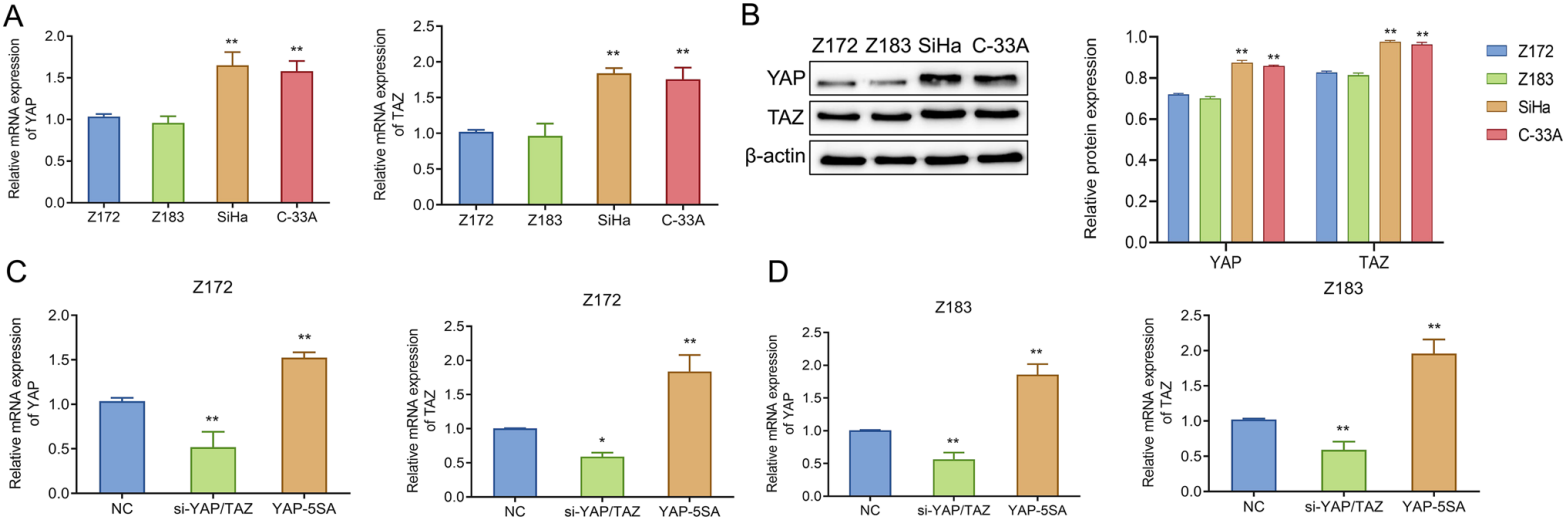
Up-regulation of YAP/TAZ expression in cervical cancer cell lines. **A** qRT-PCR to measure the mRNA expression levels of YAP and TAZ in different cervical cancer cell lines; **B** Western blot to test the protein expression levels of YAP and TAZ in different cervical cancer cell lines; **C** and **D** qRT-PCR to assess the mRNA expression levels of YAP and TAZ in Z172 (**C**) and Z183 (**D**) cells with different treatments. **P < 0.01, vs Z172, Z183 or NC. *YAP* Yes-associated protein; *TAZ* transcriptional co-activator with PDZ-binding motif

To observe the changes of YAP/TAZ expression in the progression of cervical lesions at the cellular level, qRT-PCR and western blot were applied to observe the expression of YAP/TAZ in precancerous cervical epithelial cells Z172 and Z183 and cervical cancer cells SiHa and C-33A. As shown in Fig. 2A, B, the expression levels of YAP/TAZ in Z172 and Z183 cells were significantly lower than those in SiHa or C-33A cells. To perform subsequent exploration experiments, we knocked down or over-expressed YAP/TAZ by transfection and detected the transfection efficiency by qRT-PCR. Briefly, compared with the siNC group, si-YAP/TAZ could significantly inhibit the expression levels of YAP and TAZ in Z172 and Z183 cells; while YAP-5SA remarkably increased the expression levels of YAP and TAZ in cervical cancer cell lines (Fig. 2C, D). These data indicated that the expression level of YAP/TAZ may be up-regulated in cervical cancer cell lines, thereby affecting the progress of CNI.

### YAP/TAZ promotes the stemness of precancerous cells

**Fig. 3:**
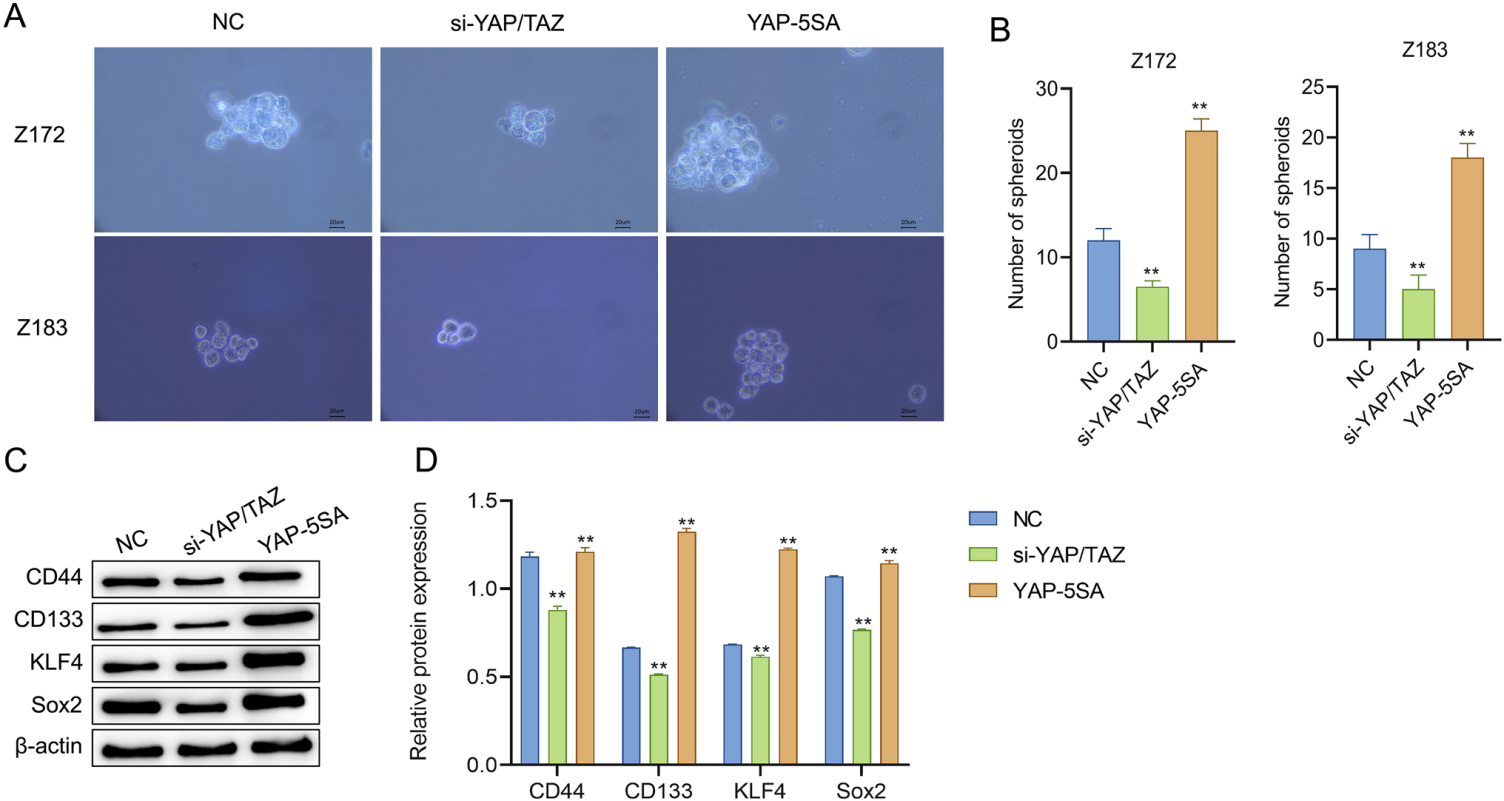
YAP/TAZ promotes the stemness of precancerous cells. **A** and **B** Sphere-formation assay to observe the spherical formation ability of Z172 and Z183 cells with different treatments (scalar bar = 20 μm). **C** and **D** Western blot to check the expression of proteins related to cancer stem cells with different treatments. **P < 0.01, vs NC

To observe the effect of YAP/TAZ expression level on precancerous cells of cervical cancer, the spherical formation ability and the expression level of cancer stem cell-related proteins in each group were checked by sphere-formation assay and western blot. Then, the effect of up-regulated YAP/TAZ expression level on CNI was further clarified. According to the results of sphere-formation assay, compared with the NC group, knock-down of YAP/TAZ significantly reduced the spherical formation ability and the number of spheres of Z172 cells and Z183 cells; while the spherical formation ability and the number of spheres of Z172 cells and Z183 cells in the YAP-5SA group increased significantly (Fig. 3A, B). The outcomes of western blot revealed that, relative to the NC group, the protein levels of CD44, CD133, KLF4 and Sox2 in Z172 and Z183 cells were notably reduced in the si-YAP/TAZ group while markedly up-regulated in the YAP-5SA group (Fig. 3C, D). In a nutshell, highly expressed YAP/TAZ promoted the stemness of Z172 and Z183 cells.

### YAP/TAZ promotes glycolysis and malignant characteristics of precancerous cells

**Fig. 4:**
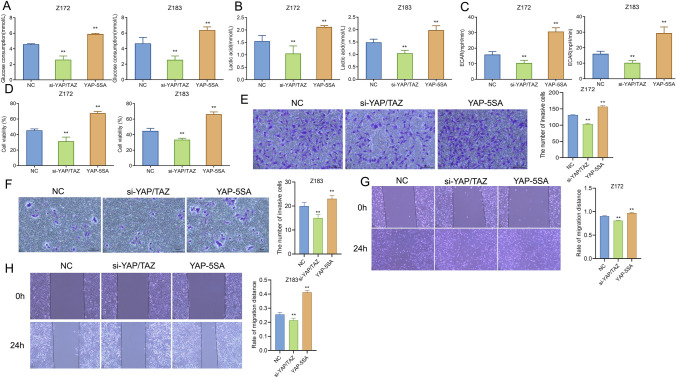
YAP/TAZ promotes glycolysis and malignant characteristics of precancerous cells. **A**–**C** The effects of different treatments on glucose consumption, lactic acid production and ECAR of Z172 and Z183 cells were observed by biochemical kits; **D** MTT assay to assess the effects of different treatments on the viability of Z172 and Z183 cells; **E** and **F** transwell to observe the impact of different treatments on the invasion ability of Z172 and Z183 cells; **G** and **H** the scratch test to observe the effects of different treatments on the migration ability of Z172 and Z183 cells. **P < 0.01, vs NC. *ECAR* extracellular acidification rate

Previous studies have stated that activating YAP/TAZ can increase the glucose uptake and glycolysis of cancer cells, promote the production of lactic acid, and result in the formation of acidic environment around cancer cells [11]. Therefore, we examined the effects of knocking down or overexpressing YAP/TAZ on glucose consumption, lactic acid production and ECAR level in precancerous cervical epithelial cell lines. Specifically, compared with the NC group, glucose consumption, lactic acid production and ECAR level of Z172 cells and Z183 cells were notably reduced after knocking down YAP/TAZ while significantly increased after over-expressing YAP-5SA (Fig. 4A–C). Additionally, the ability of cell proliferation, invasion and migration in each group was tested by MTT, Transwell and scratch test. Based on the test results, compared with the NC group, the proliferation, invasion and migration abilities of Z172 cells and Z183 cells in the si-YAP/TAZ group were significantly reduced, and undoubtedly, those in the YAP-5SA group were increased (Fig. 4D–H). The above data suggested that over-expressed YAP/TAZ promoted glycolysis and malignant characteristics of precancerous cells.

### YAP/TAZ promotes the tumorigenesis of precancerous cells in vivo

**Fig. 5:**
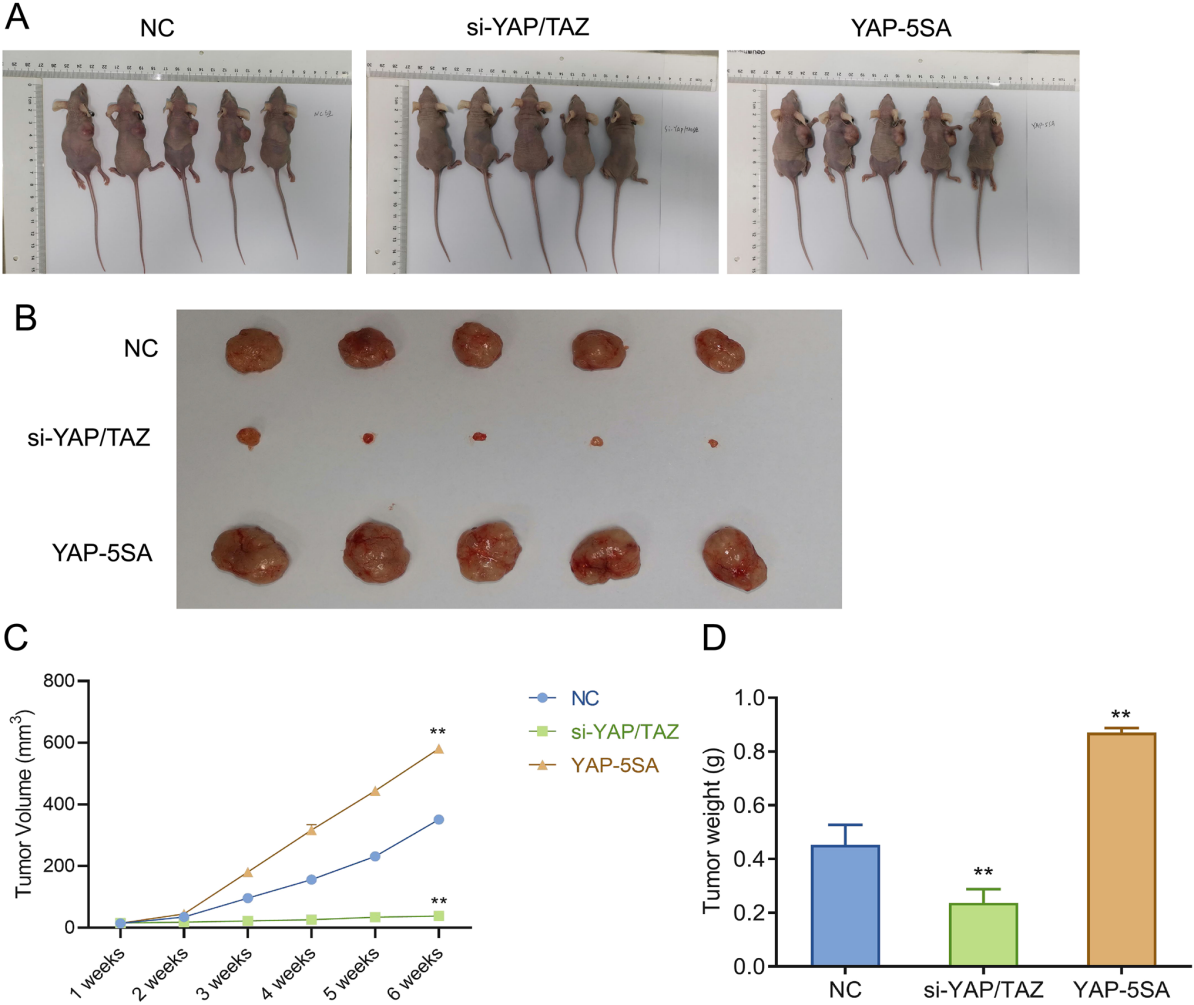
YAP/TAZ encourages the tumorigenesis of precancerous cells in vivo. **A** Tumorigenesis of rats in each group; **B** isolated tumours in each group of mice; **C** the tumor volume of mice in each group was observed every week after treatment; **D** tumor weight of mice in each group. **P < 0.01, vs si-NC

Z172 cells that knocked down or over-expressed YAP/TAZ were subcutaneously injected into nude mice to observe the effect of YAP/TAZ expression level on the tumorigenesis of precancerous cells in nude mice. In brief, relative to the NC group, the tumor grew more slowly or almost could not be observed in the si-YAP/TAZ group while increased significantly in the YAP-5SA group (Fig. 5A, B). The same phenotype was also observed in the weight difference of tumors (Fig. 5C). Subsequently, the expression of Ki67 in tumor of mice in each group was observed by immunohistochemistry. Specifically, in contrast to the NC group, the level of Ki67 decreased significantly in tumor tissue of the si-YAP/TAZ group while increased notably in that of theYAP-5SA group (Fig. 5D, E). Consequently, high expression level of YAP/TAZ promoted the tumorigenesis of precancerous cells in vivo.

### TEAD participates in the effect of YAP/TAZ on precancerous cells

**Fig. 6:**
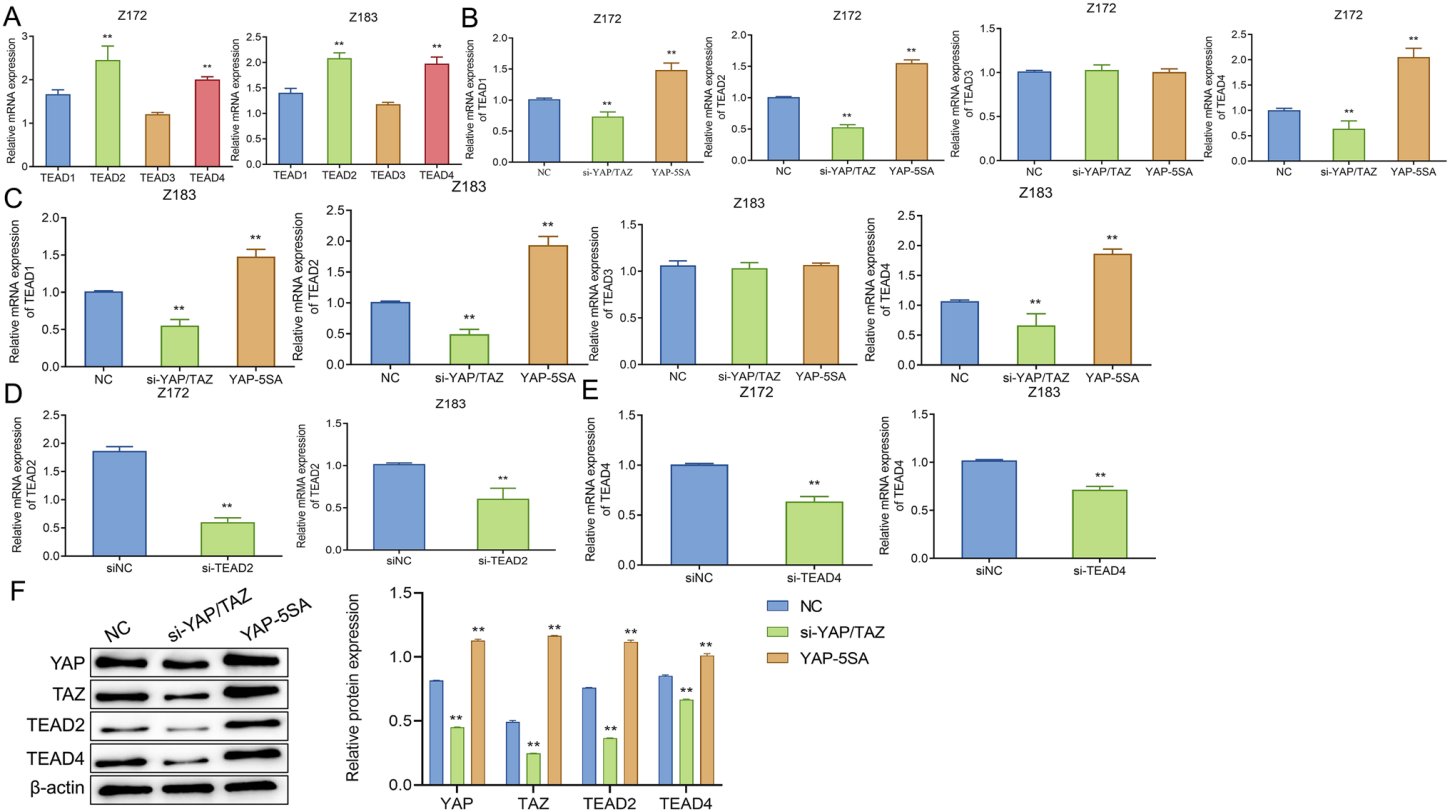
TEAD participates in the effect of YAP/TAZ on precancerous cells. **A** qRT-PCR to detect the expression levels of TEAD1, TEAD2, TEAD3 and TEAD4 in Z172 and Z183 cells, **P < 0.01, vs TEAD1 or TEAD3; **B** and **C** qRT-PCR to check the expression levels of TEAD1, TEAD2, TEAD3 and TEAD4 in Z172 (**B**) and Z183 (**C**) cells with different treatments, **P < 0.01, vs NC; **D** Western blot to observe the protein expression levels of YAP, TAZ, TEAD2 and TEAD4 after knocking down TEAD2/ TEAD4, **P < 0.01, vs si-NC; E–F, qRT-PCR to assess the expression levels of TEAD2 and TEAD4 in Z172 (**E**) and Z183 (**F**) cells after knocking down TEAD2/TEAD4. *YAP* Yes-associated protein; *TAZ* transcriptional co-activator with PDZ-binding motif; *TEAD* transcriptional enhanced associate domain

TEAD family is considered as a key participant in the intracellular transmission of YAP/TAZ signaling pathway [21]. Firstly, the expression of TEAD family proteins (TEAD1, TEAD2, TEAD3, TEAD4) in precancerous cells Z172 and Z183 was observed. Briefly, in Z172 cells, the level of TEAD2 was the highest, followed by TEAD4, TEAD1 and TEAD3; in Z183 cells, the expression levels of TEAD2 and TEAD4 were similar, but both were significantly higher than expression levels of TEAD1 and TEAD3 (Fig. 6A). Interestingly, after changing the expression level of YAP/TAZ in cells, the mRNA and protein expression levels of TEAD family genes also changed. In comparison with the NC group, the levels of TEAD1, TEAD2 and TEAD4 decreased markedly in the si-YAP/TAZ group while increased remarkably in the YAP-5SA group (Fig. 6B, C). Similar results were also observed in the level of protein (Fig. 6D). Therefore, we speculated that TEAD family genes were involved in the regulatory effect of YAP/TAZ on precancerous cells. To carry out subsequent experiments, si-TEAD2/4 was transfected into Z172 and Z183 cells and the knock-down efficiency was verified by qRT-PCR. Relative to NC cells, si-TEAD2/4 significantly reduced the expression levels of TEAD2 and TEAD4 in Z172 and Z183 cells (Fig. 6E, F). The above results suggested that the expression levels of TEAD2 and TEAD4 were successfully knocked down, and further research could be carried out.

### Knocking down TEAD2/4 inhibits the effect of YAP/TAZ on precancerous cells

**Fig. 7:**
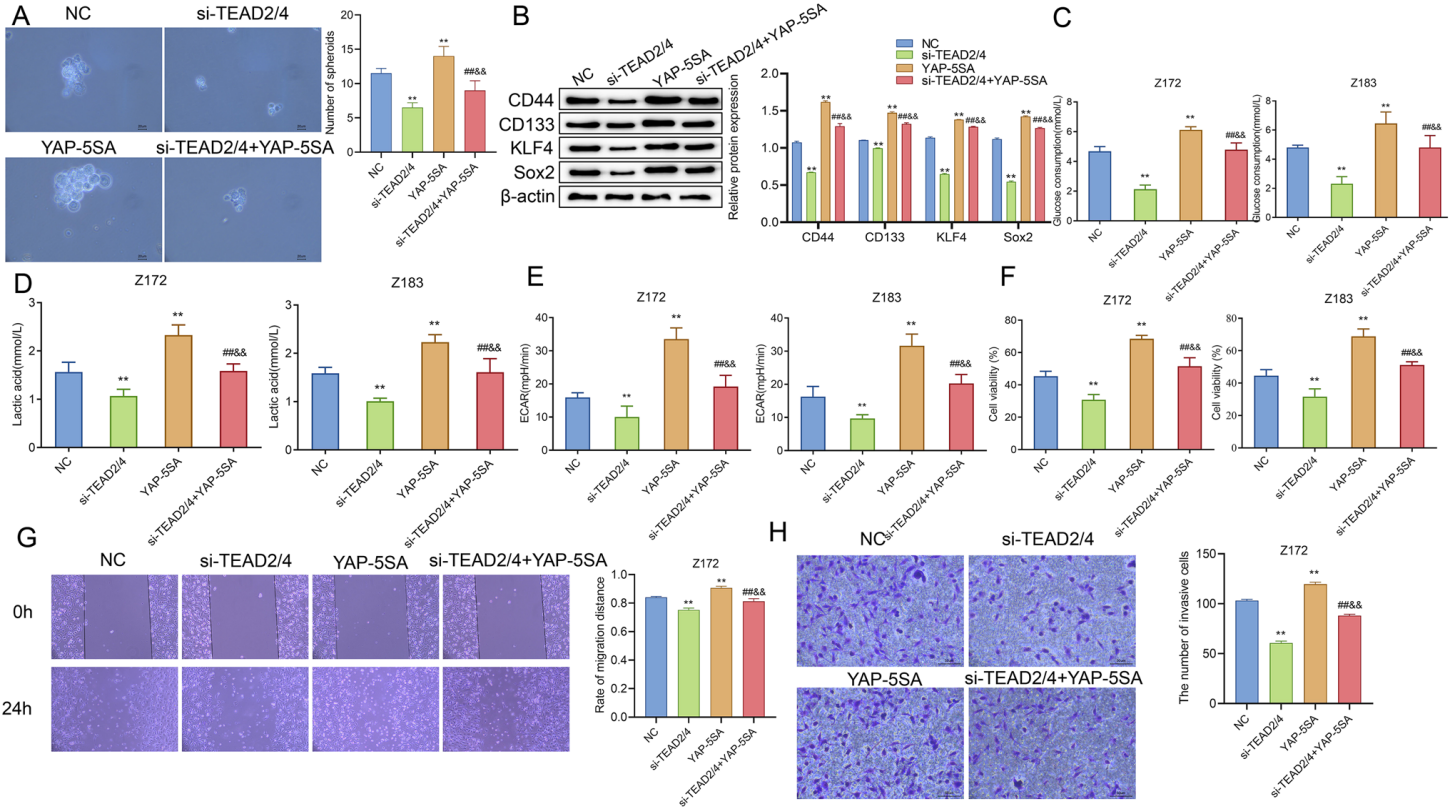
Knocking down TEAD2/4 inhibits the effect of YAP/TAZ on precancerous cells. **A** Sphere-formation assay to observe the effect of knocking down TEAD2/4 and over-expressing YAP-5SA on the spherical formation ability of cells (scalar bar = 20 μm); **B** Western blot to check the effect of knocking down TEAD2/4 and over-expressing YAP-5SA on the expression of proteins related to cancer stem cells; **C**–**E** effects of knocking down TEAD2/4 and over-expressing YAP-5SA on the glucose consumption, lactic acid production and ECAR in Z172 and Z183 cells; **F**–**H** the effect of knocking down TEAD2/4 and over-expressing YAP-5SA on the viability, invasion and migration of Z172 and Z183 cells was observed by MTT (**F**), Transwell (**G**) and scratch assay (**H**). **P < 0.01, vs NC group; ^##^P < 0.01, vs si-TEAD2/4 group; ^&&^P < 0.01, vs YAP-5SA group. *TEAD* transcriptional enhanced associate domain; *YAP* Yes-associated protein; *TAZ* transcriptional co-activator with PDZ-binding motif

In previous studies, TEAD2/4 affected the biological function of cancer cells [22], but its effect on CIN remains unknown. Therefore, the relationship between TEAD2/4 and YAP/TAZ in precancerous cells was further explored taking the spherical formation ability, viability, invasion and migration of cells as phenotypes. To be specific, after knocking down TEAD2/4 and over-expressing YAP/TAZ, the spherical formation ability of cells and the expression level of proteins related to cancer stem cells were observed. The results showed that compared with the YAP-5SA group, the spherical forming ability, the number of spheroids and the expression level of proteins related to cancer stem cells in the si-TEAD2/4+YAP-5SA group were significantly decreased (Fig. 7A, B). Also, the glycolysis level of cells in each group was observed. The observation results revealed that, compared with the YAP-5SA group, the glucose consumption, lactic acid production and ECAR level of cells in the si-TEAD2/4+YAP-5SA group were also significantly decreased (Fig. 7C–E). In addition, we also observed the malignant characteristics of cells in each group. According to the observation results, compared with the YAP-5SA group, the proliferation, invasion and migration of cells in the si-TEAD2/4+YAP-5SA group were significantly decreased (Fig. 7F–H). Hence, knocking down TEAD2/4 could inhibit the effect of over-expression of YAP/TAZ on precancerous cells.

### YAP/TAZ-TEAD activity promotes malignant transformation of precancerous cells

**Fig. 8:**
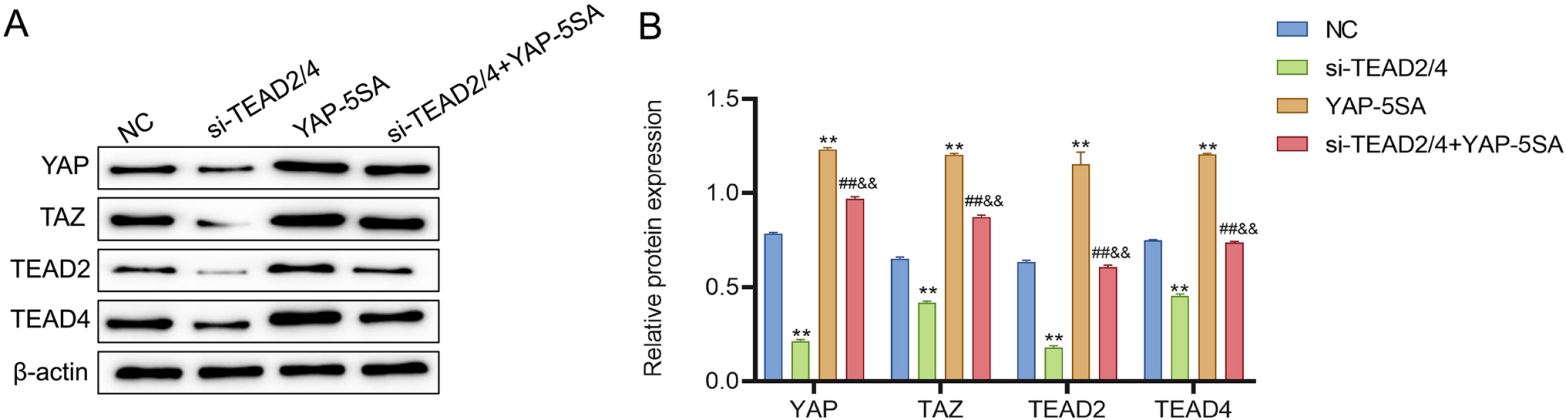
YAP/TAZ-TEAD activity promotes malignant transformation of precancerous cells. **A** and **B** Western blot to check the expression of proteins related to YAP/TAZ-TEAD in cells with different treatments. **P < 0.01, vs NC

Previous studies have stated that the regulatory effect of YAP/TAZ pathway on cells depends on TEAD23 [23], but its mechanism in CIN is still unclear. After activation of YAP/TAZ and knock-down of TEAD2/4, the expression of YAP/TAZ-TEAD pathway-related proteins in cells was observed in this study. The western blot results displayed that, compared with the NC group, the protein levels of YAP, TAZ, TEAD2 and TEAD4 in Z172 cells were notably down-regulated after knocking down TEAD2/4 while markedly up-regulated after over-expressing YAP/TAZ. Notably, relative to the si-TEAD2/4 group, the protein levels of YAP, TAZ, TEAD2 and TEAD4 in the si-TEAD2/4+YAP-5SA group were significantly increased; however, compared with the YAP-5SA group, the level of these proteins in the si-TEAD2/4+YAP-5SA group decreased significantly (Fig. 8A, B). Overall, YAP/TAZ regulated the progress of CIN by activating TEAD.

## Discussion

CIN is the most common precancerous lesion of cervical cancer, including mild, moderate and severe (CIN1/2/3). As the most common invasive cervical cancer [24], CIN will develop into SCC if it is not treated in time. Although the implementation of cervical cancer screening program has significantly reduced the incidence of cervical cancer, the prevalence of CIN is still high [25], and further measures are needed to increase the sensitivity of diagnosis. Here, we proved vital functions of YAP/TAZ-TEAD2/4 on the development of CIN.

In previous studies, YAP/TAZ was abnormally up-regulated in many human malignant tumors, fibrosis and cancer [26, 27]. Thereinto, there is a report that highly expressed YAP/TAZ and its localization in the nucleus are closely associated with the progress and prognosis of many tumors [28]. In this study, the expression level of YAP/TAZ increased with the increase of CIN grade. Moreover, the expression level of YAP/TAZ in precancerous cells was lower than that in SSC cells. Such results suggested that YAP/TAZ may be one of the key factors to promote the progress of CIN. However, it is worth noting that Chen et al. also discovered that the over-expression of TAZ could inhibit the apoptosis of cervical cancer cells and promote tumorigenesis [29]. Some studies have revealed that the expression level of YAP/TAZ in myeloma, lymphoma and leukemia is significantly reduced [30–32]. Furthermore, high YAP expression level is associated with improving the prognosis and survival rate of patients with multiple myeloma or acute myelogenous leukemia [31, 32]. Therefore, YAP/TAZ was knocked down or over-expressed in precancerous cells to explore the influence of YAP/TAZ expression level on the development of CIN. Among them, we chose to over-express YAP-5SA in cells because YAP-5SA lacks mutants of LATS phosphorylation sites of S61, S109, S127, S164 and S381, and these mutants are used to activate YAP stably and usually applied in the related research of YAP [33, 34]. In subsequent research, we observed that activation of YAP/TAZ signal could significantly increase the spherical formation ability of cells and the expression of cancer stem cell markers. In previous studies, YAP/TAZ signals were continuously activated in healthy differentiated cells in vitro. As a result, healthy differentiated cells were transformed into cells similar to tissue-specific stem cells [35], and cancer non-stem cells were transformed into cancer stem cells [36]. Although the mechanism of YAP/TAZ for reprogramming of stem cells is not clear, YAP/TAZ-driven reprogramming of stem cells has been observed in breast cells and hepatocytes [35, 37]. Such outcomes suggested the possibility that YAP/TAZ-mediated reprogramming is involved in multiple tumorigenesis.

Additionally, the effect of YAP/TAZ signal on glycolysis and malignant characteristics of precancerous cells was also observed in this study. Specifically, YAP/TAZ signal could significantly promote the glycolysis level and the malignant characteristics of cells in vitro and in vivo. According to previous studies, tumor cells mainly rely on glycolysis to provide energy and substances needed for continuous cell proliferation, even under normal oxygen conditions, which is called Warburg effect [38]. Interestingly, Wang et al. proved that YAP could promote the glycolysis of tumor cells by directly regulating the transcription of GLUT3 [11]. Besides, Song et al. discovered that YAP activation specifically increased the mRNA and protein expression levels of hexokinase 2 (HK2) [21]. Overall, YAP/TAZ signal can maintain Warburg effect of precancerous cells. In addition, YAP/TAZ participates in cell mitosis and DNA replication, promotes cell cycle and maintains the expression of oncogene transcription factors, such as activator protein 1 (AP-1) [14] and Skp2 [39], through direct transcription and induction of cancer cell lines. Moreover, activation of YAP/TAZ signal can promote the spread of non-metastatic cancer cells in vivo, while inactivation of YAP/TAZ hinders metastasis [40]. Warburg effect is one of the important metabolic markers of cancer cells. Glycolysis of cancer cells is enhanced even under aerobic conditions. Warburg effect was found to be abnormally activated in the process of tumor occurrence and metastasis, which led to the increase of glycolysis flux, activated Yap/Tead signal transduction, resulted in the activation of a series of transcription factors, and then drove the transformation of epithelial cells into interstitial cells [41]. Collectively, the activation of Warburg effect promoted the up-regulation of YAP/TAZ expression, and participated in the malignant transformation of cervical precancerous cells and the metastasis of cancer cells.

In the subsequent research, the involvement of TEAD in the regulation of YAP/TAZ on precancerous cells was further proved. After over-expressing YAP/TAZ and knocking down the expression of TEAD in precancerous cells, we observed that knocking down TEAD significantly inhibited the effect of over-expressing YAP/TAZ on precancerous cells. In the process of exploring the regulation of HK2 activity by YAP/TAZ signal, Song et al. revealed that YAP activated HK2 through binding to TEAD [21]. Besides, Gao et al. believed that the complex of YAP/TEAD could also bind to the promoter to activate HK2 transcription, thereby promoting glycolysis of breast cancer cells [23]. Moreover, the review by Hu et al. proposed the function of TEAD in promoting cancer cell proliferation and metastasis [13]. Consequently, TEAD may be the key hub that YAP/TAZ signal promotes the development of CIN.

However, there are many shortcomings in this research. On the one hand, the functions of YAP/TAZ-TEAD in different levels of CIN are not refined. On the other hand, there is no more direct evidence that activating YAP/TAZ can accelerate the transition from CIN to SCC. Therefore, further research is required to better provide data support for clinical treatment and diagnosis of cervical cancer.

## Conclusion

**Fig. 9:**
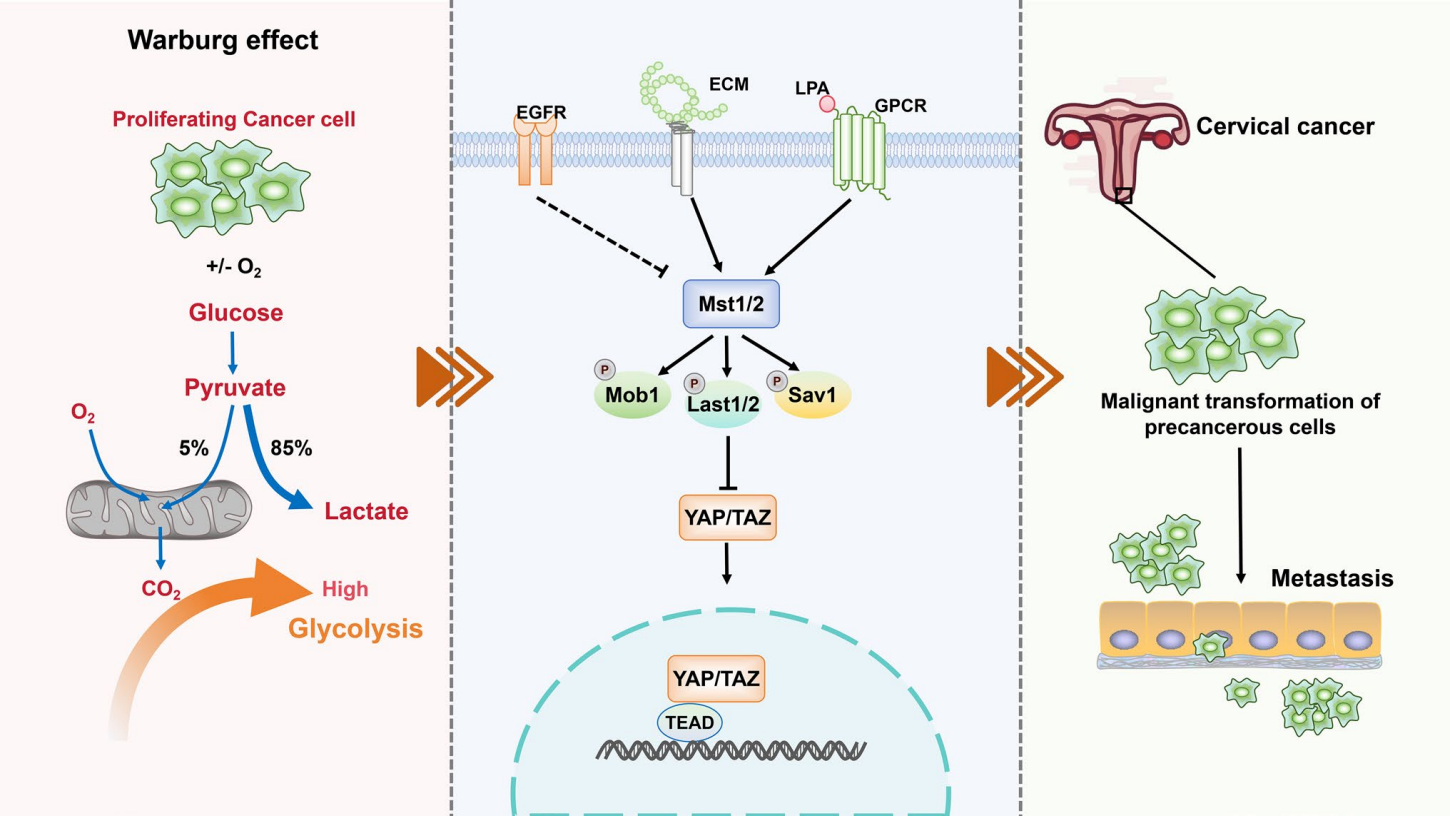
Mechanism diagram. Activation of Warburg effect in precancerous cervical epithelial cells increased the dryness and glycolysis of precancerous cervical epithelial cells, promoted the expression of YAP/TAZ signal transduction TEAD, and thus accelerated the malignant transformation of cervical intraepithelial neoplasia into cervical cancer and promoted the metastasis of cancer cells

To sum up, the activity of YAP/TAZ-TEAD increases with the development of CIN and plays a decisive role in the development of CIN. Besides, it not only increases the stemness of precancerous cells but also promote glycolysis and malignant characteristics. Therefore, the increase of Warburg effect in CIN cells promotes the transduction of TEAD expression by YAP/TAZ signal, thereby accelerating the malignant transformation of CIN into cervical cancer (Fig. 9).

## Data Availability

All related data and materials are available from the corresponding author upon request.
